# The Relationship Between Perfectionism and Sleep Quality Among Master’s Students: The Mediating Role of Competitive Personality and the Moderating Effect of Resilience

**DOI:** 10.3390/bs16050749

**Published:** 2026-05-12

**Authors:** Xiuwei Liu, Yingchun Wang, Yimeng Zhai

**Affiliations:** School of Psychology, Beijing Sport University, No. 48 Information Road, Beijing 100084, China; liuxiuwei1116@bsu.edu.cn (X.L.); zym11@bsu.edu.cn (Y.Z.)

**Keywords:** perfectionism, sleep quality, competitive personality, resilience, master’s students

## Abstract

Background: In increasingly competitive academic environments, highly perfectionistic master’s students frequently report sleep problems. The mechanisms underlying this association, however, remain unclear. This study tested whether competitive personality mediates the perfectionism–sleep quality relationship and whether resilience moderates this mediated pathway. Methods: In a cross-sectional survey, 469 master’s students (Mage = 23.23 years, 53.5% male) completed validated measures of perfectionism, competitive personality, resilience, and sleep quality. Moderated mediation analyses (PROCESS Model 15) with 5000 bootstrap resamples were conducted. Results: Perfectionistic concerns and perfectionistic strivings both positively predicted competitive personality and were associated with poorer sleep quality. Resilience moderated the second stage of the mediation pathway. The indirect effects of both perfectionism dimensions via competitive personality on sleep quality were significant at low and moderate levels of resilience, but became non-significant at high resilience (index of moderated mediation = −0.001 for concerns, −0.003 for strivings; 95% *CI*s excluded zero). Conclusions: Competitive personality acts as a key mechanism linking perfectionism to poor sleep among master’s students. High resilience buffers this indirect pathway. Strengthening resilience while targeting perfectionistic cognitions may be a promising strategy for protecting sleep quality in competitive academic settings.

## 1. Introduction

Graduate education is a critical period for academic and career development. It is also marked by intense academic pressure, demanding research expectations, and uncertainty about the future. These conditions make master’s students a high-risk group for mental health difficulties (Parkhouse et al., 2026; SenthilKumar et al., 2023). Among various health indicators, poor sleep quality has emerged as a particularly prominent concern (J. Liu et al., 2024). Poor sleep quality, which refers to dissatisfaction with sleep including aspects such as latency, duration, efficiency, and disturbances (Buysse et al., 1989), not only impairs cognitive functioning and emotional regulation but also exacerbates symptoms of anxiety and depression, creating a vicious cycle (He et al., 2025). A large-scale meta-analysis has consistently shown that perfectionistic concerns are significantly associated with poorer sleep quality among students (Stricker et al., 2023). Despite this evidence, the psychological mechanisms linking perfectionism to sleep disturbances remain underexplored, particularly in master’s students. Previous research has primarily examined rumination and anxiety as mediators, while overlooking the role of competitive personality.

According to the Dual-Process Model of Perfectionism (Slade & Owens, 1998), perfectionism consists of two higher-order dimensions: perfectionistic strivings (associated with adaptive goal pursuit) and perfectionistic concerns (characterized by excessive self-criticism, fear of failure, and a perceived discrepancy between standards and performance). These dimensions are often moderately to strongly correlated, and both can relate to competitiveness (Lizmore et al., 2019; Roy et al., 2023). Perfectionistic concerns, in particular, are consistently linked to maladaptive outcomes such as rumination and pre-sleep hyperarousal (Küskens et al., 2024; Stricker et al., 2022). However, the pathway from perfectionistic concerns to poor sleep is unlikely to be direct; it probably operates through intermediate psychological processes. Competitive personality—a disposition to strive for superiority and to avoid inferiority in social comparisons (Houston et al., 2002)—is one such mediator. Distinct from general achievement motivation or social comparison orientation, competitive personality specifically captures the tendency to compete with others in evaluative settings (Xie et al., 2006). Master’s students routinely face highly competitive environments (e.g., limited publication opportunities, fellowships, and academic posts). Perfectionistic concerns may amplify a competitive personality as a maladaptive coping strategy, leading to chronic activation, reduced pre-sleep detachment, and ultimately poorer sleep quality.

Not all perfectionistic and highly competitive individuals develop severe sleep problems. Individual differences in resilience—the ability to bounce back from adversity and regulate negative emotions—may buffer this process (Chang et al., 2023). Resilience can alter risk appraisal, cognitive responses to perceived emotions, and coping strategy selection (Kumpfer, 2002). In the present context, resilience may weaken the strength of the association between competitiveness and sleep disturbances, thereby serving as a protective factor. The current study therefore examined whether competitive personality mediates the relationship between perfectionism (concerns and strivings) and sleep quality among master’s students, and whether resilience moderates this mediating pathway.

### 1.1. The Direct Association Between Perfectionism and Sleep Quality

A growing body of research demonstrates that the two perfectionism dimensions relate differently to sleep outcomes. Perfectionistic concerns are consistently associated with poorer sleep quality, whereas perfectionistic strivings typically show weak or non-significant associations (Stricker et al., 2023). For instance, in a sample of Sudanese medical students, Elbadawi et al. (2025) found that the dimensions of “concern over mistakes and doubts about actions” and “concern over parental expectations and evaluation” significantly predicted poor sleep quality. A laboratory polysomnography study showed that higher perfectionism, particularly concern over mistakes, was associated with increased nocturnal awakenings during the first night of sleep in the laboratory (Johann et al., 2017). Actigraphy-based research also indicates that socially prescribed perfectionism predicts lower objectively measured sleep efficiency, with stress mediating the relationship (Oh et al., 2024). Among Chinese adolescents, maladaptive perfectionism has been found to affect sleep quality indirectly through worry and rumination; adaptive perfectionism not only lacks a negative effect but may also have its potential benefits suppressed in the presence of rumination (Lin et al., 2017). However, contrasting evidence suggests the role of perfectionistic strivings is more nuanced. A study of young athletes found that high perfectionistic strivings buffered the negative impact of poor sleep habits on academic and athletic performance, pointing to a possible protective function of this dimension (Roy et al., 2023). The influence of adaptive and maladaptive perfectionism on sleep is thus not unidirectional.

Cognitive appraisal theory explains part of the direct link: individuals with high perfectionistic concerns tend to interpret ambiguous or mildly stressful situations as threats rather than challenges, activating sustained cognitive arousal and pre-sleep rumination (Küskens et al., 2024). This heightened cognitive activation prolongs sleep onset and reduces overall sleep efficiency. In addition, maladaptive perfectionism exacerbates dysfunctional beliefs about sleep and low sleep self-efficacy, which further impairs sleep quality (Akram et al., 2019; Dautovich et al., 2021).

While perfectionistic concerns are consistently linked to poorer sleep, the role of perfectionistic strivings appears context-dependent. Although generally considered adaptive, perfectionistic strivings may exert different effects depending on the population and environmental demands. Graduate students operate in a particularly high-pressure academic environment characterized by sustained performance evaluation, intense peer comparison, and high self-expectations. Under these conditions, even the pursuit of high personal standards can become a source of chronic stress rather than a protective factor, potentially contributing to sleep disturbances. Moreover, perfectionistic concerns such as fear of failure and excessive self-criticism are likely amplified in this population, further impairing sleep. Therefore, we hypothesized that both dimensions of perfectionism would exhibit a significant positive direct association with poorer sleep quality among master’s students (Hypothesis 1), consistent with meta-analytic and contextual evidence (Küskens et al., 2024; Stricker et al., 2023).

**Hypothesis** **1** **(H1).** *Both dimensions of perfectionism would exhibit a significant positive direct association with poorer sleep quality among master’s students.*

### 1.2. The Mediating Role of Competitive Personality

Competitive personality refers to a tendency to strive to outperform others in the pursuit of personal growth, realizing one’s potential, or achieving a specific goal (Xie et al., 2006). In the competitive landscape of graduate education, master’s students frequently encounter peer comparison, pressure to publish, and career uncertainty. Competitive personality therefore emerges as a core psychological stressor. Although few studies have directly tested competitive personality as a mediator between perfectionism and sleep quality, indirect evidence supports its role in the stress-transmission process.

Perfectionism has been shown to enhance competitive performance in real-world settings (Stoll et al., 2008). In a laboratory competitive task, Klein et al. (2020) found that trait perfectionism independently predicted competitive performance, motivation, and strategy use. Moreover, different perfectionism dimensions appear to predict distinct forms of competitiveness. Wang and Li (2008) reported that, among college students, concern over mistakes and high expectations predicted hyper-competitiveness, whereas high personal standards and organization predicted competitive self-growth. These findings suggest that perfectionistic strivings and concerns may differentially orient individuals toward competitive contexts.

Competitive personality may transmit the effects of perfectionism to sleep. According to the stress-transmission model, perfectionistic individuals, particularly those high in perfectionistic concerns, tend to interpret competitive situations as threats. This heightens physiological and psychological arousal, thereby disrupting sleep initiation and maintenance (Richardson & Gradisar, 2020). Perfectionistic concerns increase sensitivity to social evaluation and fear of failure, both core components of competitive pressure (Elbadawi et al., 2025). In a sample of adolescents, Lin et al. (2017) found that repetitive negative thinking mediated the relationship between maladaptive perfectionism and sleep quality. Although that study did not directly measure competitive personality, competitive self-evaluation often triggers such cognitive perseveration. Furthermore, research on socially prescribed perfectionism indicates that its association with sleep difficulties is stronger among individuals with lower functional disability and higher social participation (Clementi et al., 2021), suggesting that engagement in competitive environments may amplify the detrimental effects of perfectionism on sleep.

Notably, the perfectionism–competitive personality relationship may not be uniformly positive. Stoeber (2015) found in a sample of 229 university students that self-oriented perfectionism was negatively correlated with competitiveness orientation, although unrelated to cooperativeness. This implies that perfectionistic strivings may sometimes reduce competitive striving, potentially attenuating the indirect effect on sleep. Nevertheless, given the high-pressure, evaluative context of graduate education, competitive personality remains a plausible mediator. We therefore hypothesized that competitive personality mediates the relationship between perfectionism and sleep quality (Hypothesis 2), consistent with evidence linking perfectionism to competitive orientation and competitive arousal to sleep disruption (Klein et al., 2020; Richardson & Gradisar, 2020).

**Hypothesis** **2** **(H2).** *Competitive personality mediates the relationship between perfectionism and sleep quality.*

### 1.3. The Moderating Role of Resilience

Resilience refers to the capacity to withstand, adapt to, and recover from adversity and stress (Chmitorz et al., 2018). Graduate students represent a particularly vulnerable population: they face sustained academic pressure, demanding research expectations, social comparison, and career uncertainty—all stressors that may cumulatively erode sleep quality (J. Liu et al., 2024; Parkhouse et al., 2026). For these individuals, resilience may serve as a critical buffer against the negative psychological and physiological consequences of perfectionism and competitive pressure.

Empirical evidence supports the protective role of resilience in sleep outcomes across various populations. In a sample of 479 postgraduate researchers, Milicev et al. (2021) found that resilience was the only factor consistently associated with all mental health indicators, underscoring its unique importance in academic populations. Du et al. (2020) demonstrated in a seven-country study of 2254 college students that higher resilience attenuated the adverse effects of perceived stress and anxiety on sleep quality. Similarly, Ping Chuang et al. (2024) reported that high resilience weakened the positive associations between psychological distress, rumination, and poor sleep. Feng et al. (2025) showed that resilience moderated the relationship between reflective thinking and sleep quality, the detrimental impact of reflective thinking being stronger among students with lower resilience. Among young Chinese athletes, mental toughness—a construct closely related to resilience—partially mediated the negative effect of perfectionistic concerns on sleep (Chen et al., 2024). Martínez et al. (2026) found that higher resilience was associated with better sleep quality and partially mediated the rumination–sleep association, with rumination decreasing as resilience increased. Longitudinal evidence further indicates a unidirectional protective effect: Topçu et al. (2026) reported that baseline resilience predicted improved sleep quality over time in adolescents.

Despite these established protective effects, no study to date has directly examined whether resilience moderates the perfectionism–sleep quality pathway or the competitive personality–sleep quality pathway in master’s students. Existing research on perfectionism and sleep has focused on mediators such as rumination, anxiety, depression, and dysfunctional sleep cognitions (Akram et al., 2019; Küskens et al., 2024; Lin et al., 2017), largely overlooking resilience as a potential buffer. Because perfectionistic concerns and competitive personality operate through cognitive arousal, threat appraisal, and stress generation (Elbadawi et al., 2025; Richardson & Gradisar, 2020), and because resilience is known to mitigate precisely these mechanisms (Du et al., 2020; Feng et al., 2025), it is theoretically plausible that resilience attenuates the adverse effects of both perfectionism and competitive personality on sleep. Specifically, resilience may moderate the second stage of the mediation by enabling individuals to regulate competitive arousal before it disrupts sleep. Its influence on the direct perfectionism–sleep pathway may be less pronounced, as that path captures more automatic cognitive patterns (e.g., dysfunctional sleep beliefs).

Therefore, we propose the following hypotheses:

**Hypothesis** **3a** **(H3a).** *Resilience moderates the direct relationship between perfectionism and sleep quality among master’s students, such that the positive association is weaker for individuals with higher resilience (Du et al., 2020; Feng et al., 2025).*

**Hypothesis** **3b** **(H3b).** *Resilience moderates the second stage of the mediation pathway—that is, the relationship between competitive personality and sleep quality—such that the positive association is attenuated at higher levels of resilience (Chen et al., 2024; Ping Chuang et al., 2024).*

**Figure 1:**
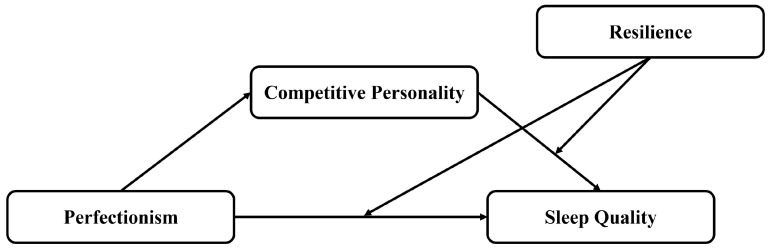
Hypothetical model diagram.

A visual representation of the proposed moderated mediation model is presented in Figure 1.

## 2. Materials and Methods

### 2.1. Participants and Procedure

This study employed a cross-sectional, questionnaire-based survey design. Participants were master’s students recruited from six universities located across eastern, western, southern, and northern China. Recruitment was conducted via online advertisements posted in graduate student WeChat groups and mailing lists. Inclusion criteria were: full-time master’s student, aged 20–30 years. The survey was administered on 23 December 2025, via www.wjx.cn, with embedded attention-check items. Completion took approximately 20–30 min. A total of 542 initial responses were received. Data were excluded based on the following criteria: (1) missing data on more than 5% of all items or omission of three or more items in any single scale (*n* = 23); (2) failure of two or more attention-check items (*n* = 18); (3) ten or more consecutive identical responses or clearly patterned responses (*n* = 12); and (4) values exceeding ±3 standard deviations from the mean (*n* = 20). After data screening, 469 valid questionnaires remained, comprising 251 males (53.5%) and 218 females (46.5%), with a mean age of 23.23 years (*SD* = 1.83). Academic disciplines included science, engineering, humanities, and social sciences. The study protocol was approved by the Ethics Application Form for Psychological Research at the School of Psychology, Beijing Sport University (approval No. 20251220).

### 2.2. Measures

#### 2.2.1. The Chinese Frost Multidimensional Perfectionism Scale (CFMPS)

The Chinese Frost Multidimensional Perfectionism Scale (CFMPS) was used to measure perfectionism. The original scale was developed by Frost et al. (1993) and cross-culturally adapted for Chinese samples Zi and Zhou (2006). It is a 27-item measure with five dimensions: Concern over Mistakes, Parental Expectations, Personal Standards, Organization, and Doubts about Actions. Items are rated on a 5-point Likert scale. Based on the dual-process model and recent recommendations (Stricker et al., 2022), Concern over Mistakes and Doubts about Actions were combined to index perfectionistic concerns, while Personal Standards indexed perfectionistic strivings. Confirmatory factor analysis indicated acceptable model fit (χ^2^/df = 4.03, *RMSEA* = 0.08, *CFI* = 0.90, *TLI* = 0.90). The internal consistency coefficient for the total scale was high (α = 0.89), and subscale alphas ranged from 0.72 to 0.90.

#### 2.2.2. The Chinese Version of the Pittsburgh Sleep Quality Index (CPSQI)

The Chinese version of the Pittsburgh Sleep Quality Index (PSQI) was utilized to evaluate participants’ sleep quality during the previous month. The original PSQI was developed by Buysse et al. (1989); its Chinese adaptation and validation were conducted by X. C. Liu et al. (1996). The PSQI consists of 19 self-rated items, of which only the first 18 are scored. It yields seven component scores—subjective sleep quality, sleep latency, sleep duration, habitual sleep efficiency, sleep disturbances, use of sleep medication, and daytime dysfunction—each ranging from 0 to 3. The sum provides a total sleep quality score ranging from 0 to 21, with higher scores indicating worse sleep quality. In the current study, the CPSQI demonstrated acceptable internal consistency (α = 0.89).

#### 2.2.3. Cooperative and Competitive Personality Scale (CCPS)

The Competitive Personality Scale, a subscale of the Cooperative and Competitive Personality Scale (CCPS), was used to measure competitive orientation. Developed by Xie et al. (2006) for Chinese populations, it is a 10-item measure comprising three dimensions: self-growth, surpassing others, and hyper-competitiveness. Items are rated on a 5-point Likert scale. Total scores are calculated as the mean across items, with higher scores indicating a stronger competitive orientation. Although the scale has multiple facets, the subscales were highly correlated in this sample, and the total score demonstrated good internal consistency (α = 0.84), supporting its use as a composite index. Confirmatory factor analysis indicated good model fit (χ^2^/df = 3.50, *RMSEA* = 0.07, *CFI* = 0.94, *TLI* = 0.92).

#### 2.2.4. The Chinese Version of the Connor–Davidson Resilience Scale (CCD-RISC)

The Chinese version of the Connor–Davidson Resilience Scale (CCD-RISC) was utilized to measure resilience. The original scale was developed by Connor and Davidson (2003); its Chinese adaptation and validation were conducted by Yu and Zhang (2007). It is a 25-item measure with three dimensions: Tenacity, Strength, and Optimism. Items are rated on a 5-point Likert scale. The total score is computed as the sum of all items, ranging from 0 to 100, with higher scores indicating greater resilience. Consistent with prior research with Chinese samples, the total score was used as a unidimensional index of resilience. Confirmatory factor analysis indicated good model fit (χ^2^/df = 3.31, *RMSEA* = 0.07, *CFI* = 0.94, *TLI* = 0.93). The internal consistency coefficient for the total scale was high (α = 0.94).

### 2.3. Statistical Analysis

Descriptive statistics, correlation analyses, and common method bias testing were performed using SPSS 26.0. A moderated mediation model was tested using the PROCESS macro (Model 15). PROCESS Model 15 was selected because it allows the moderator (resilience) to simultaneously influence the direct path from perfectionism to sleep quality and the second stage of the mediation (competitive personality → sleep quality), which corresponds directly to our hypothesized model. Prior to analysis, all continuous variables were standardized to facilitate interpretation of coefficients across different metrics. The bootstrap resampling method was applied with 5000 iterations, and a 95% confidence interval (95% CI) excluding zero was considered indicative of a statistically significant mediating effect. Simple slope analysis was conducted to examine the moderating role of resilience, for which participants were divided into low (*Mean* − 1*SD*), mean, and high (*Mean* + 1*SD*) groups for illustrative purposes. Johnson–Neyman regions of significance were also computed. Two-tailed *p* < 0.05 were considered statistically significant. Assumption checks indicated no multicollinearity (all *VIF* < 2) and approximately normal residuals. A post hoc power analysis using G-Power (version 3.1.9.6) indicated that with N = 469 and seven predictors, the study had greater than 95% power to detect a small effect size (f^2^ = 0.05) at α = 0.05.

## 3. Results

### 3.1. Common Method Bias Test

Given this study’s exclusive reliance on self-report questionnaires, the potential for common method bias was carefully examined. To assess this, Harman’s single-factor test was applied: all questionnaire items were entered into an exploratory factor analysis with the extraction constrained to a single unrotated factor. Common method bias was considered present if this single factor explained more than 40% of the total variance. The analysis identified 16 factors with eigenvalues greater than 1, and the first factor accounted for only 17.07% of the total variance—well below the 40% threshold. Thus, common method bias is unlikely to pose a significant concern in this study.

### 3.2. Correlation Analysis

Pearson correlations among the study variables are presented in Table 1. Perfectionistic concerns were positively correlated with perfectionistic strivings (*r* = 0.637, *p* < 0.01), PSQI score (*r* = 0.351, *p* < 0.01), and competitive personality (*r* = 0.386, *p* < 0.01), and negatively correlated with resilience (*r* = −0.104, *p* < 0.05). Perfectionistic strivings were positively correlated with PSQI score (*r* = 0.236, *p* < 0.01), competitive personality (*r* = 0.442, *p* < 0.01), and resilience (*r* = 0.197, *p* < 0.01). PSQI score was positively correlated with competitive personality (*r* = 0.196, *p* < 0.01) and negatively correlated with resilience (*r* = −0.105, *p* < 0.05). Competitive personality was positively correlated with resilience (*r* = 0.208, *p* < 0.01).

In summary, higher perfectionistic concerns were associated with stronger competitive personality, poorer sleep quality, and lower resilience. Higher perfectionistic strivings were associated with stronger competitive personality and poorer sleep quality, yet also with higher resilience. Competitive personality was positively associated with poorer sleep quality, whereas resilience was negatively associated with PSQI scores, indicating that more resilient students reported better sleep quality. These findings provide preliminary support for the hypothesized relationships.

### 3.3. The Moderated Mediation Model

To examine whether competitive personality mediates the relationship between perfectionism dimensions (concerns and strivings) and sleep quality, and whether resilience moderates the indirect path, two moderated mediation models (Model 15; Hayes, 2015) were tested using PROCESS macro for SPSS with 5000 bootstrap samples. PSQI total score (higher scores indicate worse sleep quality) served as the outcome variable (Y). Competitive personality was the mediator (M), resilience was the moderator (W), and gender and age were included as covariates. All continuous variables were mean-centered prior to analysis. Two separate models were run with perfectionistic concerns and perfectionistic strivings as the independent variable (X), respectively.

#### 3.3.1. Model with Perfectionistic Concerns as the Predictor

**Figure 2:**
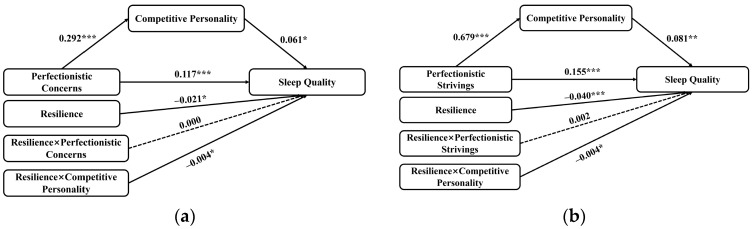
Moderated mediation model. (**a**) Moderated mediation model for Perfectionistic Concerns and PSQI; (**b**) Moderated mediation model for Perfectionistic Strivings and PSQI. *** *p* < 0.001; ** *p* < 0.01; * *p* < 0.05.

Regression results. As shown in Table 2 and Figure 2, Perfectionistic concerns significantly predicted competitive personality (β = 0.292, *SE* = 0.034, *t* = 8.645, *p* < 0.001), explaining 15.7% of its variance. In the outcome model, perfectionistic concerns had a significant direct positive effect on PSQI (β = 0.117, SE = 0.020, *t* = 5.864, *p* < 0.001). Competitive personality also positively predicted PSQI (β = 0.061, SE = 0.025, *t* = 2.480, *p* = 0.014). The interaction between competitive personality and resilience was significant (β = −0.004, SE = 0.002, *t* = −2.396, *p* = 0.017, ΔR^2^ = 0.011), indicating that the effect of competitive personality on sleep quality depended on resilience. The interaction between perfectionistic concerns and resilience was not significant (*p* = 0.880), suggesting that the direct effect of perfectionistic concerns on PSQI did not vary with resilience.

Conditional direct effects. As presented in Table 3, the direct effect of Perfectionistic concerns on PSQI was significant at low (mean −1 SD = −12.865; effect = 0.114, *SE* = 0.030, *p* < 0.001), mean (effect = 0.117, *SE* = 0.020, *p* < 0.001), and high (mean +1 SD = 12.865; effect = 0.119, *SE* = 0.021, *p* < 0.001) levels of Resilience, remaining relatively stable across conditions. Thus, higher perfectionistic concerns consistently predicted worse sleep quality regardless of resilience level.

Conditional indirect effects. As presented in Table 3, the indirect effect via competitive personality was significant at low resilience (effect = 0.034, 95% *CI* [0.015, 0.053]) and at mean resilience (effect = 0.018, 95% *CI* [0.004, 0.033]), but not at high resilience (effect = 0.002, 95% *CI* [−0.018, 0.020]). The index of moderated mediation was −0.001 (95% *CI* [−0.004, −0.002]), confirming significant moderated mediation.

**Figure 3:**
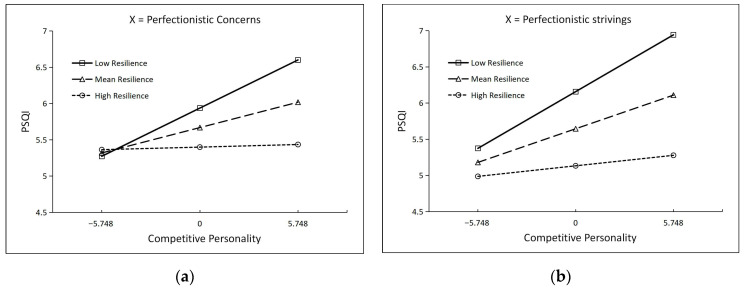
Simple Slope Plots for the Moderating Effect of Resilience on the Competitive Personality → PSQI Path. (**a**) X = Perfectionistic Concerns; (**b**) X = Perfectionistic Strivings.

Conditional effects of Competitive Personality on PSQI. Simple slope analysis (see Figure 3) revealed that showed that the competitive personality → PSQI path was significant at low resilience (effect = 0.116, *SE* = 0.037, *t* = 3.132, *p* = 0.002, 95% *CI* [0.043, 0.188]) and at mean resilience (effect = 0.061, *SE* = 0.025, *t* = 2.480, *p* = 0.014, 95% *CI* [0.013, 0.109]), but non-significant at high resilience (effect = 0.006, *SE* = 0.030, *t* = 0.208, *p* = 0.835, 95% *CI* [−0.052, 0.065]). The Johnson–Neyman technique identified that this effect was significant when resilience values were below 3.229 (58.8% of the sample) and non-significant above this threshold.

#### 3.3.2. Model with Perfectionistic Strivings as the Predictor

Regression results. As shown in Table 4 and Figure 2, Perfectionistic strivings significantly predicted competitive personality (β = 0.679, *SE* = 0.066, *t* = 10.278, *p* < 0.001), explaining 20.3% of its variance. In the outcome model, perfectionistic strivings had a significant direct positive effect on PSQI (β = 0.155, *SE* = 0.039, *t* = 3.999, *p* < 0.001), and competitive personality positively predicted PSQI (β = 0.081, SE = 0.025, *t* = 3.216, *p* = 0.001). The competitive personality × resilience interaction was significant (β = −0.004, SE = 0.002, *t* = −2.252, *p* = 0.025, ΔR^2^ = 0.010). The perfectionistic strivings × resilience interaction was not significant (*p* = 0.431).

Conditional direct effects. As presented in Table 5, the direct effect of perfectionistic strivings on PSQI was significant at low (effect = 0.124, *SE* = 0.056, *p* = 0.028), mean (effect = 0.155, *SE* = 0.039, *p* < 0.001), and high (effect = 0.186, *SE* = 0.054, *p* = 0.001) levels. Higher Perfectionistic strivings consistently predicted worse sleep quality across all resilience levels.

Conditional indirect effects. As presented in Table 5, the indirect effect via competitive personality was significant at low resilience (effect = 0.093, *BootSE* = 0.025, 95% *CI* [0.045, 0.142]) and mean resilience (effect = 0.055, *BootSE* = 0.018, 95% *CI* [0.020, 0.091]), but not at high resilience (effect = 0.017, *BootSE* = 0.023, 95% *CI* [−0.027, 0.063]). The index of moderated mediation was −0.003 (*BootSE* = 0.001, 95% *CI* [−0.005, −0.001]), confirming a significant moderated mediation.

Conditional effects of Competitive Personality on PSQI. Simple slope analysis (see Figure 3) showed that the competitive personality → PSQI path was significant at low resilience (effect = 0.136, *SE* = 0.039, *t* = 3.528, *p* < 0.001, 95% *CI* [0.060, 0.213]) and mean resilience (effect = 0.081, *SE* = 0.025, *t* = 3.216, *p* = 0.001, 95% *CI* [0.031, 0.130]) and non-significant at high resilience (effect = 0.025, *SE* = 0.031, *t* = 0.806, *p* = 0.421, 95% *CI* [−0.036, 0.087]). The Johnson–Neyman threshold was 6.933, above which the effect was non-significant (77.2% of the sample below this value).

#### 3.3.3. Summary of Findings

The results revealed the following: First, both perfectionistic concerns and perfectionistic strivings were directly associated with poorer sleep quality (higher PSQI scores), supporting H1. Second, these direct effects were not moderated by resilience; thus, H3a was not supported. Third, competitive personality partially mediated the relationships between perfectionism dimensions and sleep quality, such that higher levels of perfectionism predicted higher competitive personality, which in turn predicted worse sleep quality, supporting H2. Fourth, resilience significantly moderated the second stage of the indirect pathway (competitive personality → sleep quality). Specifically, the mediating role of competitive personality was significant among individuals with low to moderate resilience, but this indirect effect became non-significant when resilience was high, and the strength of the indirect effect decreased as resilience increased. These findings support H3b. Collectively, these results indicate that resilience serves as a protective factor that specifically buffers the detrimental pathway from competitive personality to sleep disturbances, thereby attenuating the indirect effect of perfectionism on sleep quality. Notably, the direct effects of perfectionism on sleep quality remained robust across all levels of resilience, confirming that resilience primarily buffers the mediated path rather than the direct path.

## 4. Discussion

This study constructed a moderated mediation model to investigate the mechanisms linking perfectionism to sleep quality among master’s students, focusing on the mediating role of competitive personality and the moderating role of resilience. The findings offer a nuanced understanding of how dispositional tendencies interact with environmental pressures to influence sleep health.

### 4.1. The Direct and Indirect Effects of Perfectionism on Sleep Quality

Consistent with Hypothesis 1 and the prior literature, both perfectionistic concerns and strivings were positively associated with poorer sleep quality. This pattern fits the dual-process model of perfectionism (Slade & Owens, 1998), which holds that perfectionistic strivings can be adaptive in some contexts but maladaptive under high pressure and evaluative threat. The direct association aligns with meta-analytic evidence that perfectionistic concerns are robustly linked to poor sleep (Stricker et al., 2023) and extends this work by showing that even perfectionistic strivings—the “adaptive” dimension—predicted worse sleep in graduate students. This resonates with findings that the effects of perfectionistic strivings are context-dependent (Roy et al., 2023). In the high-stakes, chronically evaluative environment of graduate education, the relentless pursuit of high standards may generate sustained cognitive activation, pre-sleep rumination, and an inability to detach from academic goals (Küskens et al., 2024; Stricker et al., 2022).

Beyond direct effects, competitive personality significantly mediated the relationship between both perfectionism dimensions and sleep quality, supporting Hypothesis 2 and the stress-transmission model (Richardson & Gradisar, 2020). Perfectionistic individuals, especially those high in concern over mistakes, tend to interpret competitive situations as threats to self-worth, heightening arousal that disrupts sleep (Elbadawi et al., 2025; Johann et al., 2017). Our findings extend the work of Klein et al. (2020), who found that trait perfectionism predicted competitive performance and motivation, by demonstrating that this competitive orientation has downstream health consequences. Both concern over mistakes and strivings for high standards appear to fuel a competitive personality, which keeps individuals in a state of hyperarousal and social comparison even during the pre-sleep period. Notably, Wang and Li (2008) found that different perfectionism dimensions predict different forms of competitiveness (hypercompetitive vs. self-growth). Our composite measure of competitive personality captured a general orientation that was uniformly detrimental to sleep in this sample, suggesting that any form of heightened competitiveness may tax sleep resources. Nevertheless, the indirect effects were small in absolute magnitude, implying that other mediators (e.g., rumination, stress) are also likely at play.

### 4.2. The Protective Moderating Role of Resilience: Buffering the Indirect Pathway

A key finding is that resilience moderated the competitive personality → sleep quality stage of the mediation but did not moderate the direct perfectionism–sleep link. This supports Hypothesis 3b but not Hypothesis 3a. The indirect effect of perfectionism on sleep via competitive personality was significant among students with low and moderate resilience but became non-significant at high resilience. The Johnson–Neyman analysis further showed that competitive personality predicted sleep problems only when resilience fell below a certain threshold.

This pattern refines our understanding of resilience as a protective factor. Rather than attenuating the direct link between a distal trait (perfectionism) and sleep, resilience operates by disrupting the translation of a proximal maladaptive state (competitive personality) into sleep disturbance. This is consistent with the view of resilience as a dynamic capacity to recover from adversity and regulate negative emotions (Chmitorz et al., 2018; Kumpfer, 2002). Highly resilient individuals, even when they possess a competitive personality, are likely equipped with superior coping strategies, such as cognitive reappraisal and problem-focused coping, which prevent competitive arousal from escalating into pre-sleep cognitive hyperarousal (Chen et al., 2024; Feng et al., 2025). In contrast, low-resilience individuals lack these buffers, and competitive personality becomes a potent stressor that directly impairs sleep. Our findings align with those of Du et al. (2020), who reported that resilience weakened the stress–sleep relationship, and with Martínez et al. (2026), who found that resilience mediated the rumination–sleep link. We extend this work by demonstrating that resilience moderates a personality-based pathway to sleep quality.

Why did resilience not moderate the direct perfectionism–sleep pathway? Perfectionism likely associates with sleep through multiple mechanisms, some of which may be less amenable to buffering by general resilience. For example, perfectionistic concerns are directly linked to dysfunctional sleep-related cognitions and low sleep self-efficacy (Akram et al., 2019; Dautovich et al., 2021), which may impair sleep regardless of resilience level. The direct path may also capture more automatic, physiological aspects of hyperarousal that are less easily modulated by conscious strategies. Resilience therefore appears to be a specific rather than a global buffer: it effectively interrupts the socially evaluative, competitive stress pathway but is less effective at altering the direct, more ingrained cognitive–behavioral patterns of perfectionism.

### 4.3. Theoretical and Practical Implications

Theoretical contributions. First, this study integrates perfectionism, competitive personality, resilience, and sleep quality into a single moderated mediation model, thereby extending the dual-process model of perfectionism (Slade & Owens, 1998) into the sleep domain. We demonstrate that the maladaptive side of perfectionism operates not only directly but also indirectly through competitive personality, and that this indirect pathway is conditional on resilience. Second, by showing that resilience moderates the competitive personality–sleep link but not the direct perfectionism–sleep link, we provide a more precise understanding of resilience’s protective boundary conditions. This challenges the oversimplified view that resilience is a universal panacea and instead suggests it works through specific mechanisms. Third, our findings contribute to the growing literature on graduate student mental health (J. Liu et al., 2024; Parkhouse et al., 2026) by identifying modifiable psychological processes in a high-risk population. To our knowledge, this is among the first studies to examine competitive personality as a mediator and resilience as a stage-specific moderator in the perfectionism–sleep link among master’s students.

Practical implications. These findings offer actionable directions for intervention. First, university mental health services should screen for both perfectionism and competitive personality as risk factors for sleep problems. Students with high levels of these traits, especially those with low resilience, should be prioritized for preventive interventions. Second, resilience-building programs should be implemented, focusing on teaching students how to detach from competitive arousal during the pre-sleep period. Mindfulness-based stress reduction (MBSR) and cognitive–behavioral therapy for insomnia (CBT-I) could be adapted to include modules on reappraising competitive thoughts and fostering a non-judgmental attitude toward academic performance. Third, given that the direct association of perfectionism remained robust, interventions should also directly target perfectionistic beliefs (e.g., through cognitive restructuring to reduce concern over mistakes and doubts about actions) and promote self-compassion. Fourth, at the institutional level, fostering a less hypercompetitive academic culture may reduce the activation of competitive personality in the first place. Because the observed effects were modest in size and this study is cross-sectional, these practical suggestions should be viewed as preliminary and require evaluation in intervention trials.

### 4.4. Limitations and Future Directions

Several limitations must be acknowledged. First, and most importantly, the cross-sectional design precludes any causal or directional inferences. All reported associations are correlational in nature, and alternative models (e.g., poor sleep leading to increased perfectionism or competitiveness) cannot be ruled out. Longitudinal studies with multiple time points are urgently needed to establish temporal precedence and test the proposed mediation and moderation effects over time. Second, reliance on self-report questionnaires introduces the risk of common method bias and recall bias. Although Harman’s single-factor test indicated that common method bias was not a major concern, this test is only a basic diagnostic and does not rule out common method variance entirely; future research should incorporate objective measures of sleep, such as actigraphy or polysomnography, as well as informant reports or behavioral measures of competitiveness, and employ more robust techniques like the CFA marker approach. Third, our sample, while geographically diverse, consisted only of master’s students from China. Cultural specificity is an important limitation: competitiveness and resilience may be expressed and valued differently in collectivistic cultures, and the relationships observed may differ in other cultural contexts. Generalizability to undergraduate students, doctoral students, or students in other countries remains unknown. Fourth, although we included gender and age as covariates, other potential confounders—such as socioeconomic status, parenting style, baseline mental health diagnoses, or academic workload—were not measured. Future studies should include these variables. Fifth, the effects, while statistically significant, were generally small in magnitude. Future research should examine whether these small effects accumulate over time and whether they translate into clinically meaningful sleep impairment. Sixth, measurement limitations should be noted: our operationalization of perfectionism via selected subscales, the use of a composite competitive personality score, and the unidimensional treatment of resilience may obscure facet-level dynamics. Future work should differentiate between hyper-competitiveness and self-growth competition, and examine the three resilience dimensions separately.

## 5. Conclusions

Perfectionistic concerns and strivings were associated with poorer sleep quality among master’s students. Competitive personality partially mediated these associations, and high resilience attenuated the indirect pathway by weakening the competitive personality–sleep link. Resilience did not moderate the direct perfectionism–sleep association, suggesting that resilience operates as a specific protective factor in the stress-transmission process rather than as a universal buffer. Enhancing resilience, alongside directly targeting perfectionistic beliefs, may offer a promising strategy to protect sleep in competitive academic contexts, although intervention studies are needed to confirm these preliminary findings.

## Figures and Tables

**Table 1:** Correlation Matrix of Perfectionism, Competitive Personality, Resilience, and Sleep Quality.

Variables	Perfectionistic Concerns	Perfectionistic Strivings	PSQI	Competitive Personality	Resilience
Perfectionistic Concerns	1				
Perfectionistic Strivings	0.637 **	1			
PSQI	0.351 **	0.236 **	1		
Competitive Personality	0.386 **	0.442 **	0.196 **	1	
Resilience	−0.104 *	0.197 **	−0.105 *	0.208 **	1

Note: PSQI = Pittsburgh Sleep Quality Index; higher PSQI scores indicate worse sleep quality. ** *p* < 0.01; * *p* < 0.05.

**Table 2:** Regression Results for the Moderated Mediation Model (X = Perfectionistic Concerns).

Dependent Variable	Independent Variable	*R*	*R* ^2^	*F*	β(*SE*)	*t*
Competitive Personality	Perfectionistic Concerns	0.396	0.157	28.873 ***	0.292 (0.034)	8.645 ***
	Gender				−1.002 (0.497)	−2.015 *
	Age				−0.075 (0.134)	−0.557
PSQI	Perfectionistic Concerns	0.394	0.155	12.108 ***	0.117 (0.020)	5.864 ***
	Competitive Personality				0.061 (0.025)	2.480 *
	Resilience				−0.021 (0.010)	−2.102 *
	Resilience × Perfectionistic Concerns				0.000 (0.001)	0.151
	Resilience × Competitive Personality				−0.004 (0.002)	−2.396 *
	Gender				−0.262 (0.250)	−1.050
	Age				0.112 (0.067)	1.673

Note: PSQI = Pittsburgh Sleep Quality Index; higher PSQI scores indicate worse sleep quality. Gender was coded (e.g., 0 = male, 1 = female). All continuous variables were mean-centered. *** *p* < 0.001; * *p* < 0.05.

**Table 3:** Conditional Indirect, Direct Effects, and Index of Moderated Mediation (X = Perfectionistic Concerns).

Effect	Resilience Level	Effect Size	BootSE	95% BootCI
Indirect effect: Perfectionistic Concerns → Competitive → PSQI	Low (−1 SD = −12.865)	0.034	0.010	[0.015, 0.053]
	Mean (0)	0.018	0.007	[0.004, 0.033]
	High (+1 SD = 12.865)	0.002	0.010	[−0.018, 0.02]
Direct effect: Perfectionistic Concerns → PSQI	Low	0.114	0.030	[0.055, 0.173]
	Mean	0.117	0.020	[0.077, 0.156]
	High	0.119	0.021	[0.078, 0.160]
Index of moderated mediation		−0.001	0.001	[−0.004, −0.002]

**Table 4:** Regression Results for the Moderated Mediation Model (X = Perfectionistic Strivings).

Dependent Variable	Independent Variable	*R*	*R* ^2^	*F*	β(*SE*)	*t*
Competitive Personality	Perfectionistic Strivings	0.450	0.203	39.402 ***	0.679 (0.066)	10.278 ***
	Gender				−0.920 (0.483)	−1.904
	Age				−0.116 (0.130)	−0.893
PSQI	Perfectionistic Strivings	0.338	0.114	8.504 ***	0.155 (0.039)	3.999 ***
	Competitive Personality				0.081 (0.025)	3.216 **
	Resilience				−0.040 (0.010)	−3.957 ***
	Resilience × Perfectionistic Strivings				0.002 (0.003)	0.788
	Resilience × Competitive Personality				−0.004 (0.002)	−2.252 *
	Gender				−0.331 (0.257)	−1.291
	Age				0.100 (0.069)	1.450

Note: PSQI = Pittsburgh Sleep Quality Index; higher PSQI scores indicate worse sleep quality. Gender was coded (e.g., 0 = male, 1 = female). All continuous variables were mean-centered. *** *p* < 0.001; ** *p* < 0.01; * *p* < 0.05.

**Table 5:** Conditional Indirect, Direct Effects, and Index of Moderated Mediation (X = Perfectionistic Strivings).

Effect	Resilience Level	Effect Size	BootSE	95% BootCI
Indirect effect: Perfectionistic Strivings → Competitive → PSQI	Low (−1 SD = −12.865)	0.093	0.025	[0.045, 0.142]
	Mean (0)	0.055	0.018	[0.020, 0.091]
	High (+1 SD = 12.865)	0.017	0.023	[−0.027, 0.063]
Direct effect: Perfectionistic Strivings → PSQI	Low	0.124	0.056	[0.013, 0.235]
	Mean	0.155	0.039	[0.079, 0.231]
	High	0.186	0.054	[0.080, 0.291]
Index of moderated mediation		−0.003	0.001	[−0.005, −0.001]

## Data Availability

The data that support the findings of this study are available from the corresponding author upon reasonable request.
